# Persistent Hiccups as the Only Presenting Symptom of ST Elevation Myocardial Infarction

**DOI:** 10.1155/2018/7237454

**Published:** 2018-03-11

**Authors:** Nasreen Shaikh, Rishi Raj, Srinivas Movva, Charles Mattina

**Affiliations:** ^1^Department of Internal Medicine, Monmouth Medical Center, 300 Second Avenue, Long Branch, NJ 07740, USA; ^2^Department of Internal Medicine, Division of Cardiology, Monmouth Medical Center, 300 Second Avenue, Long Branch, NJ 07740, USA

## Abstract

Clinical manifestations of acute myocardial infarction can be more than just chest pain. Patients can present with dyspnea, fatigue, heart burn, diaphoresis, syncope, and abdominal pain to name a few. Our patient was a 74-year-old male with a past medical history of type 2 diabetes mellitus, hypertension, hyperlipidemia, and COPD due to chronic tobacco use, who presented with persistent hiccups for 4 days and no other complaints. Coincidently, he was found to have a diabetic foot ulcer with sepsis and acute kidney injury and hence was admitted to the hospital. A routine 12-lead EKG was done, and he was found to have an inferior wall ST elevation myocardial infarction. He underwent diagnostic catheterization which demonstrated 100% right coronary artery occlusion and a thallium viability study which confirmed nonviable myocardium; hence, he did not undergo percutaneous coronary intervention. Elderly patients who present with persistent hiccups should be investigated for an underlying cardiac etiology.

## 1. Introduction

This case report aims to spread awareness amongst the emergency department physicians and primary care physicians who often encounter patients with symptoms which seem trivial but have a grave underlying pathology. Persistent hiccups is one of them. Our case report highlights a patient who came in with similar complaints and was found to have an acute ST segment elevation myocardial infarction of the inferior wall. Persistent hiccups in the elderly with risk factors for coronary artery disease should be investigated further.

## 2. Presentation

A 74-year-old male with a past medical history of type 2 diabetes mellitus, hypertension, hyperlipidemia, and COPD from chronic tobacco use came with complaints of persistent hiccups for 4 days and no other complaints. Coincidently, he was noted to have a diabetic foot ulcer on the right great toe. On admission, the patient was afebrile with a blood pressure of 96/62 mm Hg and a heart rate of 72 beats per minute. Laboratory tests which included a complete blood count, comprehensive metabolic panel, and urinalysis were significant for a WBC count of 16 × 10^3^/*μ*L, creatinine of 1.29 mg/dL which was acute, and a lactic acid level of 2.3 mmol/L. The rest of the laboratory tests and chest X-ray were normal. Blood cultures were sent immediately and had no growth over the course of hospital stay. The admitting diagnosis was sepsis from a diabetic foot ulcer, and the patient was started on vancomycin and cefepime.

**Figure 1:**
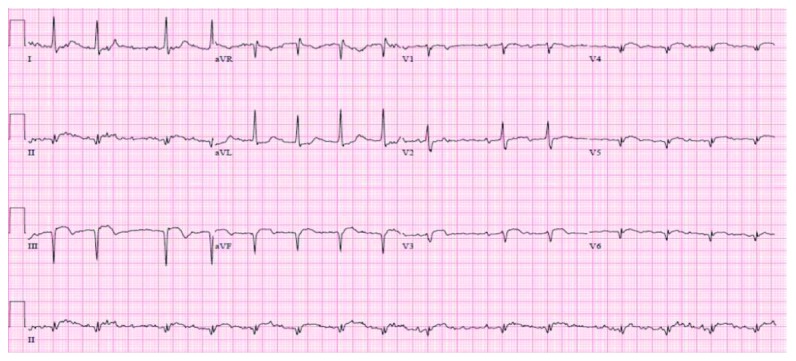
12-lead EKG showing ST elevation and Q waves in leads II, III, and aVF and sinus rhythm with first-degree AV block.

**Figure 2:**
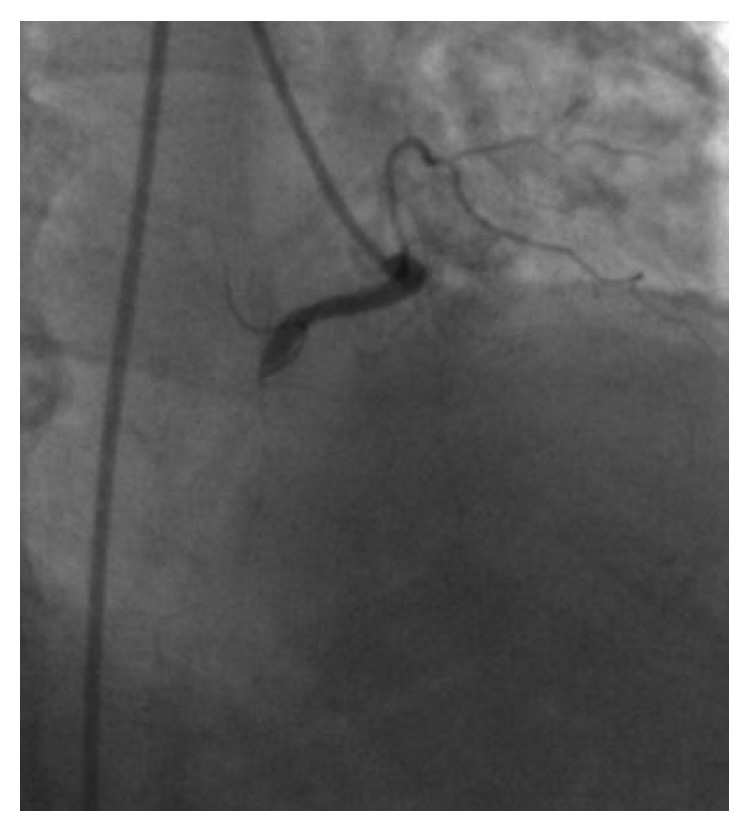
Coronary angiography showing 100% occlusion of the mid-right coronary artery.

**Figure 3:**
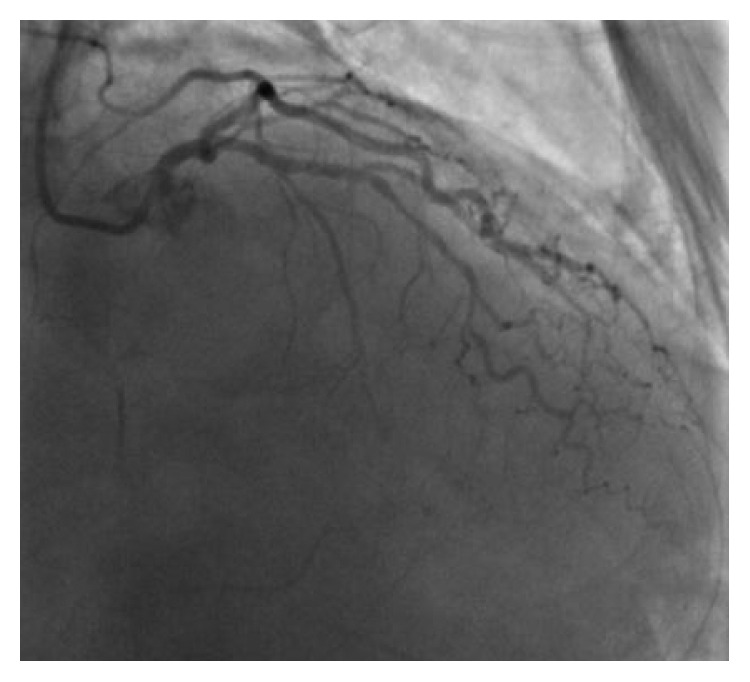
Coronary angiography showing 80% occlusion of the left anterior descending artery.

**Figure 4:**
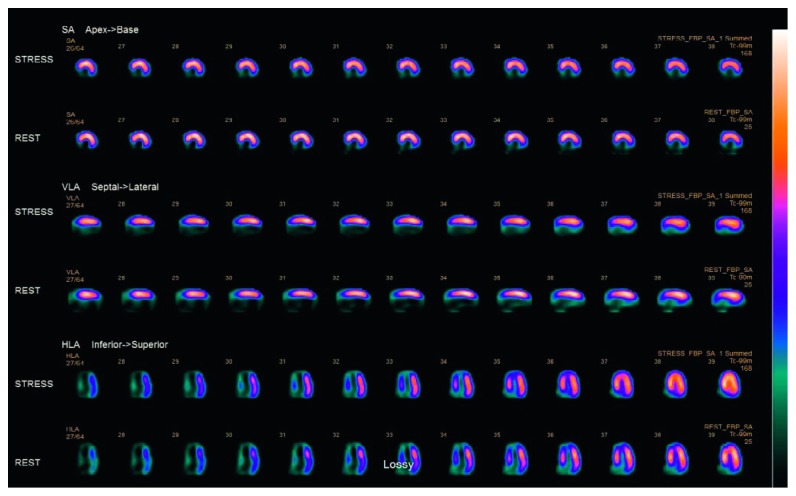
Nuclear regadenoson stress test showing inferoseptal wall defect.

Blood cultures showed no growth, and on the basis of wound cultures showing methicillin-sensitive *Staphylococcus aureus*, doxycycline was substituted. A routine 12-lead EKG was performed on admission which showed ST elevation in inferior leads with Q waves and sinus rhythm with first-degree AV block (Figure 1) with troponin-I levels of 38.22 ng/dl, suggestive of a recent age indeterminate inferior wall ST elevation myocardial infarction. He was given intravenous unfractionated heparin, aspirin, and ticagrelor. Lactic acid trended down within 4 hours. Hiccups resolved on day 2 of hospital stay, and troponins started to trend down within 6 hours of presentation. Echocardiogram showed LVEF of 30% with inferior wall akinesia. After the resolution of sepsis and acute kidney injury, diagnostic cardiac catheterization was performed which showed 100% mid-right coronary artery (RCA) occlusion (Figure 2), inferior wall akinesis, and 80% mid-left anterior descending (LAD) lesion (Figure 3), and ischemic cardiomyopathy was confirmed. A nuclear regadenoson stress test was performed which showed large inferoseptal wall defect with akinesia and surrounding anteroseptal wall hypokinesia (Figure 4). To rule out hibernating myocardium, a thallium viability scan was performed which showed a fixed inferoseptal and apical perfusion abnormality with no redistribution in 24-hour delayed imaging. As no viable tissue in the infarcted area was identified and there was no ischemic myocardium, medical therapy was recommended. The patient was discharged with an external cardiac defibrillator vest (LifeVest), and later on, he had an implantable cardiac defibrillator placed when there was no improvement in ejection fraction despite maximal medical therapy for secondary prevention of sudden cardiac death. The patient continues to do well on therapy and close follow-up.

## 3. Discussion

Our patient only complained of hiccups and displayed no other symptoms that would indicate myocardial ischemia. Although our patient did not have any other symptoms to suggest acute coronary syndrome, the patient did have multiple risk factors for coronary artery disease such as type 2 diabetes mellitus, hypertension, hyperlipidemia, and chronic smoking. “Anginal equivalent” is a term used for symptoms of acute coronary syndrome other than chest pain and includes dyspnea, fatigue, heart burn, abdominal pain, nausea, and vomiting. Acute coronary syndrome in diabetics, elderly, and female patients may present with such anginal equivalents [1]. Our patient was an elderly diabetic male who presented with a sole complaint of persistent hiccups and was found to have ST elevation myocardial infarction. Their resolution as the patient entered the convalescent stage of their AMI suggests that it was not a coincidence. None of the guidelines mention persistent hiccups as an anginal equivalent.

Hiccups are defined as sudden onset of erratic diaphragmatic and intercostal muscle contraction, immediately followed by laryngeal closure leading to abrupt rush of air into the lungs eliciting a “hic” sound. Usually, they are self-limiting, but if episodes last >48 hours, they are defined as persistent hiccups [2]. The reflex arc of the hiccup has 3 components: the afferent limb composed of phrenic, vagus, and sympathetic nerves; a central processor in the mid-brain; and the efferent limb composed of the phrenic nerve supplying the diaphragm and intercostal nerves supplying the intercostal muscle fibers [3]. Any irritants in the reflex pathway, such as physical, chemical, inflammatory, or neoplastic process, can trigger hiccups. Most commonly, persistent hiccups are caused by the nervous system disorder, either central (neoplastic and inflammatory) or peripheral by irritation of the phrenic nerve (goiter) or irritation of the vagus nerve (otolaryngologic diseases, meningitis, esophageal, stomach, and duodenal diseases, hepatitis, pancreatitis, and enteritis) [4].

This is the first case in literature in which ST segment elevation myocardial infarction presented with persistent hiccups as the sole presenting complaint. Myocardial ischemia has been reported to cause hiccups; however, there have been very few case reports in literature. Among the few reported cases, either hiccups was one of the associated symptoms in patients with myocardial ischemia or persistent hiccups presented as non-ST elevation myocardial infarction (NSTEMI). The earliest case report was in 1958, where a patient complained of dyspnea and orthopnea along with hiccups [5]. In 2 cases reported by Ikram et al. in 1971, patients developed intractable hiccups 2 to 3 days following acute inferoposterolateral and acute anterolateral MI, respectively [6]. Many other case reports cite hiccups as an associated symptom after admission for myocardial infarction [7, 8]. In a case reported by Davenport et al. in 2012, similar to our case, the patient presented with persistent hiccups without any other complaints; however, the patient was found to have inferior wall NSTEMI, and a subsequent cardiac catheterization revealed significant stenosis of the left circumflex and first obtuse marginal coronary arteries [9]. Zhang et al. reported a case of persistent hiccups with chest pain in a patient with cocaine-induced inferior wall STEMI with 99% occlusion of the mid-RCA and 80% stenosis of the LAD artery [4].

It is interesting to note that all the published literature about persistent hiccups and myocardial ischemia has identified the inferior wall of the myocardium to be affected. The inferior wall also known as the diaphragmatic surface of the heart lies in close proximity to the diaphragm. Irritation of phrenic nerves, which innervates the diaphragm, can be the cause of persistent hiccups in these patients. The other probable reason could be irritation of the vagus nerve supplying the pericardium [10].

Whether it is the inflammatory markers released from the infarcted inferior myocardium which irritate the diaphragm and trigger the hiccup reflex arc or it is the irritation of the vagus nerve directly remains to be elucidated [11]. In one of the case reports where both RCA and LAD were occluded, the symptoms of hiccups resolved after opening of only RCA even when LAD remained obstructed. This supports our observation of inferior wall MI causing hiccups [12]. This case reminds us of importance of having a high index of suspicion, especially in elderly diabetic patients where benign self-limiting condition like hiccups can be the only presenting symptoms of a serious underlying pathology like ST elevation myocardial infarction.

## 4. Conclusion

Whilst there are more common causes of acute onset persistent hiccups, if there are no other obvious causes, acute myocardial ischemia should be considered as a potential differential.
